# Cellular mechanometabolism: stimuli and implications

**DOI:** 10.1038/s44319-026-00795-4

**Published:** 2026-05-09

**Authors:** Ismael Ortiz, Santiago Lopez, Raghu Vamsi Kondapaneni, Jose V Hernandez, Keefer Boone, Cynthia A Reinhart-King

**Affiliations:** Department of Bioengineering, Rice University, Houston, TX USA

**Keywords:** Cancer, Cell Adhesion, Polarity & Cytoskeleton, Metabolism

## Abstract

While much is known about the effects of the chemical microenvironment on cellular metabolism, mechanical cues have emerged as critical stimuli of intracellular metabolic pathways. Mechanical signals from the extracellular matrix (ECM), neighboring cells, and the microenvironment intersect with key regulators of cellular metabolism, often leading to changes in fundamental cell behaviors, including cell proliferation and migration. Here, we review recent work that has uncovered a role for mechanical cues from microenvironmental factors on cellular metabolism. We discuss how cell–ECM interactions and forces such as shear, tension, and compression affect cellular metabolic requirements and energy production. Importantly, mechanometabolism shapes both physiological homeostasis and pathological states, and further investigation has implications for understanding tissue function and disease progression and uncovering potential therapeutic strategies.

## Abstract (web summary)

This review discusses recent work showing the functional impact of mechanical cues from the microenvironment on cellular metabolism in both physiological homeostasis and pathological states, expanding our understanding of mechanometabolism.

## Graphical abstract

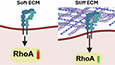

## Introduction

Cells interpret and respond to mechanical and structural cues within their surrounding microenvironment, significantly influencing fundamental cellular behaviors (Burberry et al, 2014; Chaudhuri et al, 2016; Engler et al, 2006; Janmey et al, 2020; Kim et al, 2019a; Zhou et al, 2012). Mechanical forces experienced by cells, such as compression, tension, and shear stress, can modulate diverse processes, including differentiation (Mammoto et al, 2011; Rosowski et al, 2015), proliferation, migration (Jia et al, 2020; Uroz et al, 2024; Wang et al, 2024; Zhang et al, 2015), and apoptosis (Mitchell et al, 2017; Zhao et al, 2024). The force-mediated alteration of these processes can influence the progression of various diseases, including cancer, developmental disorders, and cardiovascular conditions (Jaalouk and Lammerding, 2009). Such mechanically reprogrammed cellular activities are also coupled with changes in the metabolic activity that are necessary to sustain the increased energetic load. Consequently, mechanobiology and metabolism are fundamentally linked. The term mechanometabolism has been coined to describe the integration of mechanotransduction and metabolic regulation, whereby physical cues from the extracellular microenvironment are translated into intracellular signaling programs that reshape cellular metabolism.

Signaling from mechanical cues and cellular metabolism converge at multiple points, most prominently at cell-cell and cell–ECM adhesions and the cytoskeleton. Many classically identified mechanosensitive proteins function as metabolic regulators, positioning mechanical cues as active determinants of metabolic activity. Filamentous actin in the cytoskeleton is a dynamic scaffold for glycolytic enzymes, which enables bidirectional crosstalk between cytoskeletal remodeling and glycolytic flux (DeWane et al, 2021). In parallel, YAP/TAZ transcriptional activation has been shown to enhance glycolytic reprogramming, with perturbations to glycolytic pathways directly affecting YAP/TAZ activity as well (Zhang et al, 2018). With both cytoskeletal organization and YAP/TAZ mechanosensing tied to intracellular and extracellular mechanics, such examples highlight the active control exerted by canonical mechanotransductive proteins within cellular metabolism. As such, mechanical alterations in the extracellular environment can directly affect cellular metabolic behavior. Early links between mechanics and metabolism have been reviewed elsewhere (Romani et al, 2021). Here, we focus on how specific microenvironmental factors, including matrix stiffening, confinement, fluid shear, and compression, reprogram metabolic pathways that drive pathophysiological progression.

## ECM as a physical cue for metabolism regulation

**Figure 1:**
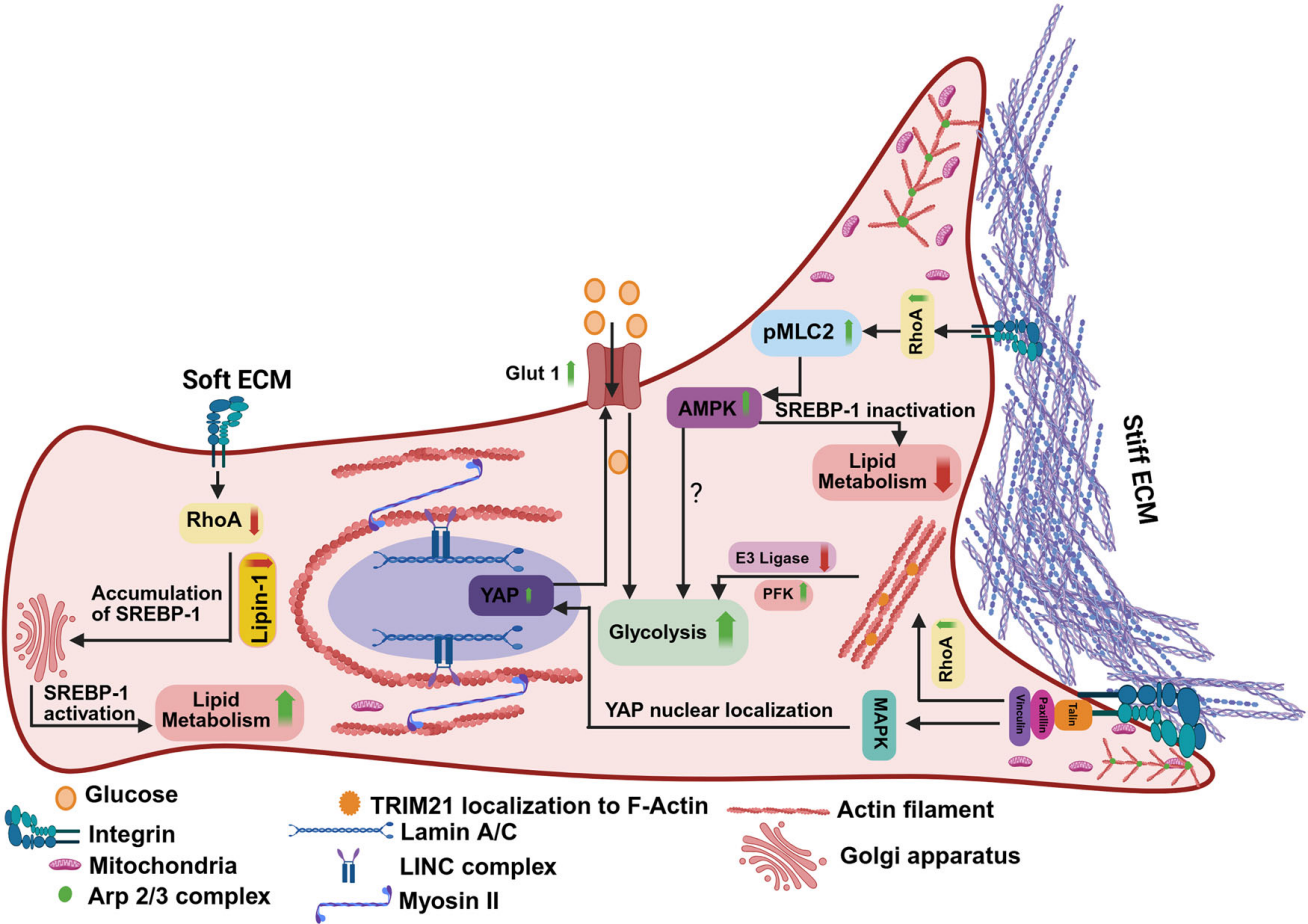
Mechanisms involved in the regulation of cellular metabolism via matrix stiffness. An increase in ECM stiffness reprograms cellular metabolism to favor glycolysis through the nuclear localization of Yes-associated protein (YAP), or by the localization of TRIM21 to F-actin bundles, and by the reduction of reliance on lipid metabolism via AMPK. YAP Yes-associated protein, PFK phosphofructokinase, MAPK mitogen-activated protein kinase, AMPK adenosine monophosphate-activated protein kinase, Glut1 glucose Transporter type 1, TRIM21 tripartite motif (TRIM)- containing protein 21, RhoA Ras homolog family member A, SREBP-1 sterol regulatory element-binding protein 1, pMLC2 phosphorylated myosin light chain 2. The green arrow indicates an increase; the red arrow indicates a decrease.

Cells are physically connected to the ECM through integrin-cytoskeletal linkages. Notably, the cytoskeleton plays a key role in both mechanotransduction and cellular metabolism. Cytoskeletal networks, including filamentous actin (F-actin), microtubules, and intermediate filaments (IFs), not only respond to cellular energy status but can also influence metabolic processes by organizing glycolytic enzymes and modulating mitochondrial function. F-actin acts as a crucial binding site for glycolytic enzymes, including phophofructokinase-1 (PFK1) (Roberts and Somero, 1989), aldolase (Ouporov et al, 1999), and GAPDH (Forlemu et al, 2011; Poglazov and Livanova, 1986). The assembly and stability of microtubules have been proposed as markers for energy sensing in cells, as exposing cells to ATP-depleted conditions leads to microtubule disassembly (Bershadsky and Gelfand, 1981; Marcussen and Larsen, 1996). Recent studies suggest a link between the length of microtubule bundles and mitochondrial fission (Mehta et al, 2019) and a relationship between ECM stiffening and microtubule glutamylation (Torrino et al, 2021). IFs are also associated with maintaining mitochondrial potential and energy flux (Mado et al, 2019), with reduced IF expression causing increased mitochondrial dysfunction and oxidative stress (Elsnicova et al, 2022; Håversen et al, 2018). Collectively, these findings establish the cytoskeleton’s role as an active metabolic scaffold, rather than a passive structural component of the cell. Given that ECM-derived mechanical and biochemical cues are transmitted into the cell via integrins and associated adhesion proteins that link the ECM to the cytoskeleton, and strong evidence suggests that the cytoskeleton affects metabolism, there is now mounting evidence that alterations to the ECM will affect the cytoskeleton and subsequently, metabolism (Fig. 1).

### Influence of ECM composition on cellular metabolism

The ECM contains a variety of adhesion proteins that have both structural and signaling roles. As the scaffolding around cells, the ECM provides and mediates mechanical cues transmitted to cells, which can modulate cellular signaling pathways and ultimately affect metabolic activity (Ponce et al, 2021; Sousa et al, 2019). When cells adhere to the ECM, they receive mechanical and biochemical cues that support metabolic processes such as glycolysis and oxidative phosphorylation (OXPHOS) via signaling pathways like PI3K-Akt-mTOR (Grassian et al, 2011). Cell–ECM adhesions mediated by integrins regulate key signaling pathways, such as the PI3K-Akt-mTOR pathway, which are central to energy homeostasis (Kang et al, 2012; Qiu et al, 2023; Wan et al, 2019). ECM adhesion also facilitates cytoskeletal organization and integrin-mediated mechanotransduction, which are essential for mitochondrial function and ATP production (Buchheit et al, 2012). Conversely, cells that lose contact with the ECM, such as during detachment or anoikis, shift toward a catabolic metabolism, undergoing autophagy or apoptosis. Detachment from the ECM also alters redox balance and mitochondrial activity, causing cells to rely more on glycolysis to limit the production of reactive oxygen species (ROS) (Mason et al, 2017).

The type of ECM ligand and ligand-integrin interaction can affect metabolism. As one example, fibronectin is a major glycoprotein present in numerous tissues to which cells can bind to form focal adhesions (FAs) (Lu et al, 2020), activating the FAK–Src–PI3K pathway. There are multiple downstream effectors, including YAP nuclear localization, which is known to regulate glucose metabolism (Kim and Gumbiner, 2015; Nardone et al, 2017; Pelaz and Tabernero, 2022). Similarly, laminins are located within the basal lamina of the ECM and bind integrins (Aumailley, 2013). The laminin matrix can increase glucose-stimulated NADPH metabolism by enhancing the expression of FGFR5 in the FGFR5/FGFR1 signaling complex, where overexpression of FGFR5 increases the NADPH/NADP+ ratio and the expression of glycolytic enzymes in beta islet cells (Pal et al, 2022). These data highlight adhesion as a metabolically instructive interface, where ligand identity affects intracellular energy pathways.

Matrix mechanics and architecture also modulate metabolism. Collagen is one of the most abundant proteins in the body. It helps maintain the tissue’s structure through its fibrous networks and multiple cross-links (Kular et al, 2014). Researchers have demonstrated that changes in collagen architecture (Zanotelli et al, 2018, 2019, 2024), and density (Zanotelli et al, 2022) significantly impact glucose metabolism and cellular energetics. Interestingly, an increase in collagen density led to decreased oxygen consumption and glucose metabolism in the tricarboxylic acid cycle in mammary carcinoma cells (Morris et al, 2016). However, if only the migratory cells within the population are studied, metabolic activity increases with collagen density, resulting in a reduction in migratory energy efficiency due to higher ATP demands in mammary adenocarcinoma MDA-MB-231 cells (Zanotelli et al, 2022). Furthermore, confinement, an architectural aspect of a dense ECM that can limit migration, biases cellular migration towards a path of least resistance, as cells placed on bifurcating collagen microtracks preferentially entered the wider branch where cellular ATP levels are reduced (Zanotelli et al, 2019). These observations suggest that alterations in collagen abundance and density can provide constraints that actively shift metabolic requirements, underscoring collagen’s role in bioenergetic regulation.

Cell-mediated ECM remodeling results in changes to ECM composition and structure that can further reshape the mechanical and metabolic landscape experienced by cells through changes in ATP utilization activity and cell-mediated ECM degradation. ECM degradation leads to reduced stiffness, which in turn reduces integrin engagement and cytoskeletal tension. This reduced engagement and tension shift the cell toward catabolic processes, such as autophagy (Jabbari et al, 2015; Liu et al, 2015). Matrix alignment and fiber organization also affect cellular metabolism. Matrix alignment has been shown to affect both migration directionality and the metabolic efficiency of migrating cells, as denser matrices impair migration, increasing ATP utilization, whereas aligned collagen matrices facilitate energy-efficient migration (Zanotelli et al, 2018, 2024). On aligned collagen, cells uptake less glucose, make less ATP, and hydrolyze less ATP. When tension is applied counter to alignment, cells migrate in the direction of tension and utilize even less ATP (Zanotelli et al, 2024). Together, these data suggest that the terrain is part of a feedback mechanism to mediate cellular energetics.

### ECM stiffness as a metabolic regulator

ECM stiffening occurs in various disease states due to matrix deposition and cross-linking (Fig. 1). An increase in ECM stiffness affects key enzymes involved in metabolic processes, including those responsible for glucose, lipid, and amino acid metabolism. Cells cultured on stiff substrates exhibit an increase in glycolysis and OXPHOS rate along with an increase in intermediate proteins involved in both processes, including fructose 6-phosphate, 3-phosphoglycerate, α-ketoglutarate, and malate (Chakraborty et al, 2021). In contrast, cells on more compliant substrates reduce their overall ATP levels (Tilghman et al, 2012). In addition, on more compliant ECM, epithelial cells transition into an energy-storing state through the accumulation of lipid droplets in the cytoplasm, mediated by the activity of Sterol Regulatory Element Binding Proteins (SREBPs). SREBPs accumulate at the Golgi apparatus in response to decreased actomyosin contractility (Bertolio et al, 2019; Romani et al, 2019). SREBP functions as an activator of lipid metabolism when the ECM is softer and cellular contractile forces are reduced. Further investigation is warranted into how ECM stiffness regulates SREBP accumulation and whether this influences other SREBP-dependent functions, such as endoplasmic reticulum and mitochondrial contact site formation, a mechanism involved in lipid synthesis (Ganji et al, 2023), as well as how different ECM components affect SREBP activity.

ECM stiffness activates the YAP/TAZ pathway and the Rho-GTPase and RHO-associated protein kinase pathways (Fig. 1) through increases in cytoskeletal tension (Nardone et al, 2017). This YAP/TAZ activation increases the expression of glucose transporters and metabolic enzymes, influencing glucose metabolism and gluconeogenesis. The YAP transcription factor has been shown to upregulate the expression of GLUT1 (Cox et al, 2018; Liu et al, 2020) and GLUT3 (Wang et al, 2015) glucose transporters. It has also been observed to upregulate glucose metabolic enzymes, including lactate dehydrogenase and hexokinase 2, through JNK and p38 signaling (Liu et al, 2020). In gluconeogenesis, phosphoenolpyruvate carboxykinase 1 and glucose-6-phosphatase catalytic subunit enzymes are repressed through the activation of the YAP/TAZ signaling pathway, which inhibits the ability of PGC1α to bind to and activate the transcription of gluconeogenic gene promoters (Hu et al, 2017). Since ECM stiffness activates YAP/TAZ, these data suggest that stiffness may increase glucose transport and reduce gluconeogenesis.

In parallel with YAP/TAZ-driven transcriptional reprogramming, ECM-induced cytoskeletal tension engages Rho-GTPase/ROCK signaling to remodel actin, thereby regulating glycolysis and broader glucose metabolism. Specifically, actin remodeling initiated by RAC1 leads to the release of the glycolytic enzyme aldolase, which is regulated by PI3K (Hu et al, 2016). Mechanical cues are generally thought to increase actin stress fiber formations, and aldolase is known to bind and release from F-actin. Therefore, ECM stiffness could play a role in mediating aldolase activity. Since mechanical cues can affect cytoskeletal structure and assembly, and numerous metabolic enzymes interact with the cytoskeleton, the field is only beginning to understand and identify these intersections.

### Fluid shear stress (FSS): impact on cellular metabolism

**Figure 2:**
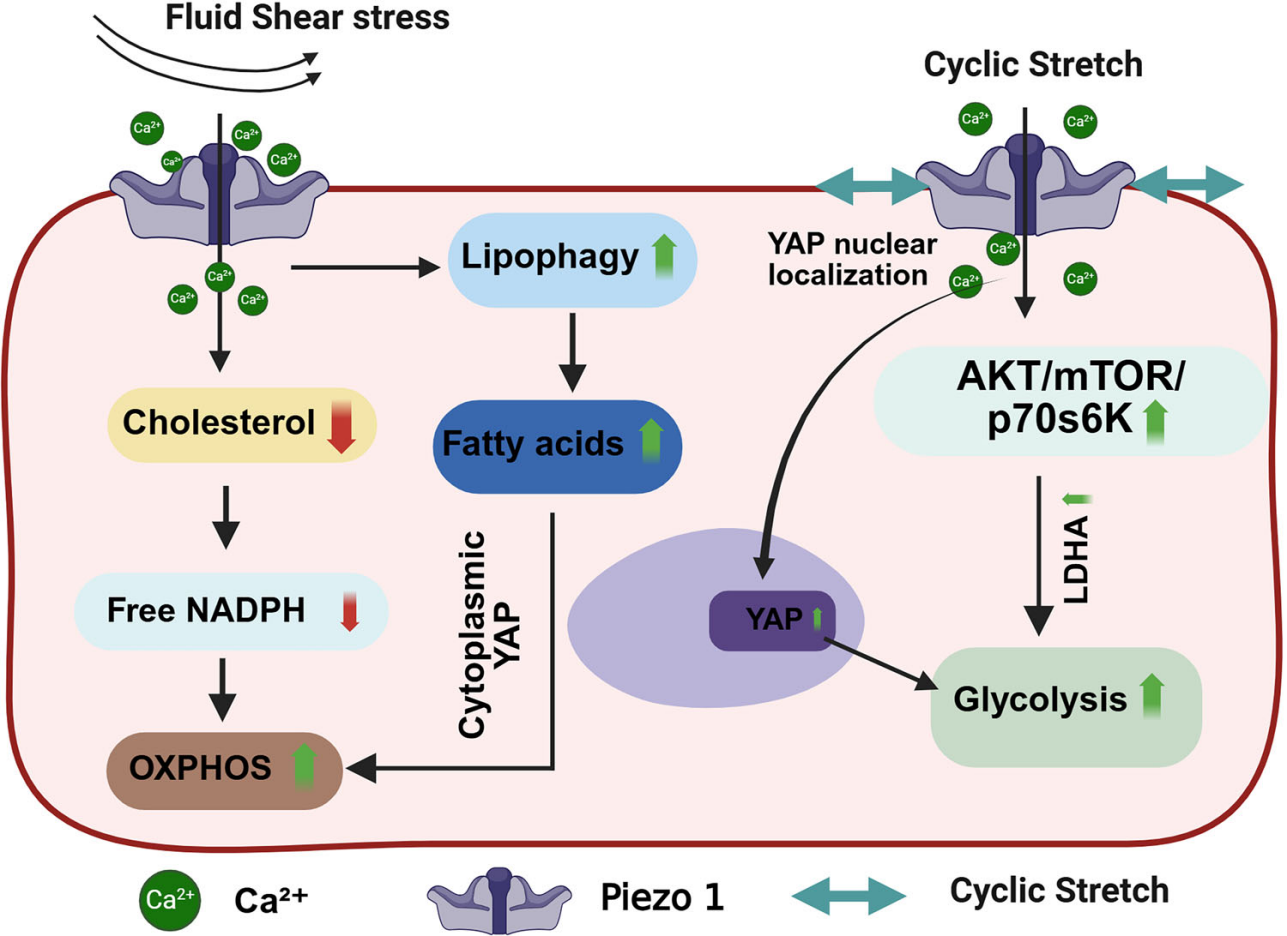
Fluid shear stress and cyclic stretch-mediated mechanisms of cellular metabolism. Under fluid shear stress, cells upregulate the piezo1 ion channel and shift their metabolism from glycolysis to OXPHOS by lowering cholesterol and free NAD(P)H levels, while increasing fatty acid levels and calcium influx. Cyclic stretch also activates the piezo1 ion channel, which then promotes glycolysis either through nuclear co-localization of YAP or by activating the AKT/mTOR/p70S6K pathway. OXPHOS oxidative phosphorylation, YAP Yes-associated protein, LDHA lactate dehydrogenase A, AKT protein kinase B, mTOR mammalian target of rapamycin, NAD(P)H nicotinamide adenine dinucleotide phosphate. The green arrow indicates an increase; the red arrow indicates a decrease.

Fluid shear stresses primarily occur in the blood and lymph systems as well as in the kidney, digestive tract, and in interstitial spaces. FSS is a key regulator of endothelial cell metabolism, significantly affecting the balance between glycolysis, OXPHOS, and overall ATP production. In human umbilical vein endothelial cells (HUVECs), cholesterol depletion induced by low laminar FSS fluidizes the plasma membrane and activates mechanosensitive receptors and ion channels, which in turn enhances mitochondrial respiration (Fig. 2) (Bodin and Burnstock, 2001; Ferreira et al, 2020; Yamamoto et al, 2020) shifting metabolism from glycolysis to OXPHOS, evidenced by reduced free NAD(P)H and diminished migration (Shen et al, 2018). Notably, laminar and oscillatory shear elicit different metabolic responses. Laminar shear causes bovine aortic endothelial cells to upregulate endothelial nitric oxide synthase (eNOS) (Moore et al, 2010), increasing nitric oxide (NO) levels, which inhibit cytochrome c oxidase (Clementi et al, 1999; Tenopoulou and Doulias, 2020; Zhou et al, 2019). As a result, mitochondrial oxygen consumption decreases, facilitating hypoxic adaptation (Groschner et al, 2012). Conversely, oscillatory shear in human aortic endothelial cells upregulates VEGFR-dependent protein kinase Cε, significantly enhancing glycolytic activity (Leung and Shi, 2022), highlighting how physiologically relevant flow regimes differentially tune endothelial metabolism to maintain homeostasis and pointing to fluid-dynamics–informed therapeutic targets for vascular dysfunction. The silencing of metabolically related proteins like HIF-1α (Wu et al, 2017) and AMPK (Yang et al, 2018) reduces glycolytic activity and increases oxidative phosphorylation even during oscillatory stress, suggesting that shear stress alone is not sufficient to maintain a glycolytic phenotype. In endothelial systems, these flow-dependent metabolic adaptations are mediated in part by the mechanically activated ion channels Piezo1 and Piezo2, which convert shear stress into Ca²⁺ influx to initiate downstream signaling cascades that couple mechanosensation to nitric oxide signaling and inflammatory activation (Fig. 2) (Albarrán-Juárez et al, 2018).

Beyond the endothelium, FSS has been shown to modulate the metabolism in other cell types: in kidney epithelial cells, for example, FSS suppresses autophagy via an AMPK-SIRT1-YAP-dependent mechanism (Claude-Taupin et al, 2023; Miceli et al, 2020a, 2020b). Osteoblasts respond to oscillatory flow signals through αvβ3/β1 integrins via FAK–Shc–PI3K and Akt–mTOR–p70S6K pathways to sustain energetically costly proliferation (Lee et al, 2010), with low shear also promoting growth-factor and adhesion-molecule expression (Bacabac et al, 2005; Liegibel et al, 2004). Within the immune compartment, Piezo1-mediated mechanotransduction links shear forces to metabolic activation states, as Ca²⁺ influx through Piezo channels primes macrophages and other immune cells for NLRP3 inflammasome activation and bioenergetic upregulation under mechanical stimulation (Fish and Kulkarni, 2024; Ran et al, 2023). In immune cells, findings suggest that treatment with consistent FSS using cone-in-plate viscometers boosts T cell activation via Piezo1, which has been shown to induce a substantial glycolytic shift aimed to support T cell proliferation and immune responses (Hope et al, 2022; Qiao et al, 2019; Sarna et al, 2024; Shyer et al, 2020; Wu et al, 2019). These Piezo-dependent mechanometabolic programs have therapeutic relevance, as failure to account for mechanical context may underestimate immune activation and metabolic vulnerabilities within tumors and inflamed tissues (Qu and Zhang, 2025), whereas excessive mechanical stimulation may exacerbate inflammatory injury (Baratchi and Peter, 2025). Understanding these mechanisms deepens our insight into cell physiology and encourages the development of innovative approaches to manipulate these responses in clinical settings. Together, these findings underscore conserved mechano-metabolic coupling across diverse cell types and motivate deeper mapping of FSS-sensing nodes to enable therapeutic manipulation in mechanically influenced diseases.

### Compression and tension: impact on cellular metabolism

Numerous tissues throughout the body are exposed to compressive and tensile forces, and these forces can also impact cellular metabolism. As an example, physiological compression within the avascular, hypoxic cartilage niche biases chondrocytes toward glycolysis to sustain ATP production under limited oxygen availability (Eitner et al, 2022; Sun et al, 2024). Compressive stress upregulates glycolytic pathways while suppressing mitochondrial respiration (Yao et al, 2019). Metabolomic profiling corroborates these shifts as shear and compression reshape chondrocyte metabolism, consistent with the adaptive reallocation of metabolic demands towards glycolysis (Welhaven et al, 2022). In primary human chondrocytes, physiological dynamic compression similarly directs central carbon metabolism toward glycolysis and the pentose phosphate pathway, increasing amino acid precursor supply to meet biosynthetic demand to create matrix (Salinas et al, 2019).

Excessive mechanical loading shifts chondrocyte bioenergetics from adaptive to pathological by elevating mitochondrial oxidants and impairing OXPHOS. Mechanical strain and overload generate oxidants in articular cartilage and raise mitochondrial superoxide while lowering the antioxidant SOD2 (Brouillette et al, 2014; Koike et al, 2015). Whereas physiologic stress couples ATP synthesis to sublethal mitochondrial oxidants under hypoxia, improper loading disrupts this coupling and diminishes respiratory efficiency, predisposing tissue to degeneration (Wolff et al, 2013). Together, these findings indicate that excessive mechanical loading shifts mitochondria from adaptive redox signaling to maladaptive oxidative dysfunction. Sustained mitochondrial dysfunction/ROS promotes chondrocyte senescence linked to osteoarthritis pathogenesis (Shao et al, 2024). Hydrostatic pressure is associated with effusion-driven intra-articular pressure in osteoarthritis and has been shown to increase ROS in cartilage pellets (Rieder et al, 2018). Cross-tissue evidence shows that scavenging mitochondrial ROS attenuates pressure-overload–induced mitochondrial proteome changes, underscoring ROS as a driver of pressure-mediated mitochondrial dysfunction (Dai et al, 2012).

Tension and cyclic stretch also reprogram cellular energetics across tissues through mechanoresponsive pathways such as YAP/TAZ and Akt–mTOR (Fig. 2). Tension and cyclic stretch occur in various organs, most prominently in the lungs, heart, and skeletal system. Such forces can profoundly influence cellular metabolism across different cell types. In osteoblasts, cyclic stretch enhances glucose metabolism by increasing lactate production and upregulating glycolysis-related enzymes via the Akt/mTOR signaling pathway (Zeng et al, 2015). When cyclic tension is applied to differentiate mesenchymal stem cells into smooth muscle cells, glycolysis, OXPHOS, and mitochondrial fusion are elevated, mediated through increased YAP nuclear localization (Liu et al, 2025). In models mimicking stretch injury in alveolar epithelial cells, cyclic stretch promotes glycolysis through the upregulation of glycolytic enzymes such as phosphofructokinase, leading to an accumulation of pyruvate and lactate (Vohwinkel et al, 2022). In addition, increased surface tension on lung epithelial cells enhances ATP release, which may regulate mucociliary clearance and surfactant secretion (Ramsingh et al, 2011). Collectively, these data establish mechanical loading as a primary governor of cellular bioenergetics. Physiologic compression biases chondrocytes toward glycolysis to sustain matrix maintenance, whereas pathologic pressure elevates mitochondrial ROS and impairs OXPHOS to promote degeneration; similarly, tensile and cyclic stretch cues reset the glycolysis-OXPHOS balance via YAP/TAZ and Akt–mTOR, underscoring load pattern and magnitude as levers to restore metabolic homeostasis.

### Mechanometabolism in the tumor microenvironment

Cancer mechanometabolism is complicated by metabolic plasticity that has made metabolic-targeting therapies difficult to create (Desbats et al, 2020; Hanahan and Weinberg, 2011; Stine et al, 2022). Metabolic rewiring also shapes cancer cell identity by regulating epigenetic modifiers and stem-like phenotypes, contributing to neoplastic progression (Saggese et al, 2020). The Warburg effect was defined many years ago to describe the metabolic shift cells undergo to support the biosynthetic requirements of uncontrolled proliferation (Warburg, 1956). However, recent studies have shown that cancer cells may employ hybrid metabolic states (Anderson et al, 2014; Jia et al, 2019; Sancho et al, 2015; Yu et al, 2017) and can dynamically adjust their bioenergetic needs in response to changes in the tumor microenvironment (TME) (Zanotelli et al, 2021). This metabolic plasticity is not purely cell-intrinsic; it is actively directed by the TME, where progressive matrix stiffening creates a spatial mechanical gradient, selectively engaging mechanotransductive pathways that steer cells towards malignant phenotypes (Bertolio et al, 2023).

Most solid tumors stiffen during progression due to increased matrix deposition and cross-linking (Baghban et al, 2020). Cancer cells sense this stiffening through mechanotransductive pathways (Nazemi and Rainero, 2020; Su and Karin, 2023) that activate YAP/TAZ and mTOR to reprogram the cell’s metabolism toward glycolysis and glutaminolysis to sustain proliferation and survival under adverse conditions (Koo and Guan, 2018; Muir et al, 2018). Mechanical cues in the TME such as solid stress, interstitial fluid pressure/flow, shear, and matrix stiffness reshape cytoskeletal tension and thereby alter metabolic pathways (Bertero et al, 2019; Ip et al, 2016; Sullivan et al, 2018; Sun et al, 2019). In solid tumors, elevated interstitial fluid pressure exposes cancer cells to sustained shear and convective nutrient transport. Such flows bias migration along pressure gradients and can modulate metabolic programs by altering nutrient availability, oxygen transport, and redox balance (Samanta, 2020; Polacheck et al, 2011). Elevated elastic modulus promotes a pro-tumor phenotype(Li et al, 2020) and activates the mTOR pathway to meet the heightened energy demands necessary for tumor growth and metastasis (Laplante and Sabatini, 2012; Zhang et al, 2024). Stiff substrates also increase glycolysis by sequestering TRIM21 in F-actin bundles and preserving the activity of phosphofructokinase (Park et al, 2020). Stiffness further augments amino acid metabolism, promoting kindlin-2 translocation into the mitochondria to facilitate proline synthesis (Guo et al, 2019) and increases MMP-9 expression, linking stiffness to proteolysis and invasion (Rainu and Singh, 2024). As such, it is important to study cancer cells within in vivo -like conditions because both the cell type and the conditions may affect response.

Beyond canonical mechanotransductive effectors, matrix stiffening also engages oncogenic programs that reshape amino acid metabolism and nutrient acquisition. Missense mutant p53 upregulates serine-glycine synthesis enzymes and the L-type amino acid transporter LAT1 to sustain proliferation under amino acid limitation (Tombari et al, 2023), while mechanical cues stabilize mutant p53 via a mevalonate–RhoA axis, enabling gain-of-function tumorigenic activities (Ingallina et al, 2018). Together, these findings reveal that stiffness not only activates YAP/TAZ and mTOR signaling but also stabilizes oncogenic drivers that rewire metabolic pathways to support tumor growth.

Notably, the mechanical relationships observed in 3D matrices often differ fundamentally from those on 2D substrates, reflecting the distinct architectural and confinement regimes intrinsic to volumetric extracellular environments. In 3D tumor stroma, more compliant or soft matrices can enhance cancer cell proliferation, invasion, and metastatic dissemination, reflecting reduced physical confinement and stromal resistance that facilitate tissue deformation and movement (Jiang et al, 2020; Fenner et al, 2014). Specifically, in breast cancer organoid models, invasive cells at the periphery are larger, softer, and more dynamic due to supracellular fluid-flow-mediated softening, indicating that permissive 3D mechanics promote invasion (Han et al, 2020). In addition, 3D matrix stiffness has been shown to induce heritable mitotic errors and chromosomal alterations (Anlaş et al, 2025), highlighting a mechanics-driven evolution of solid tumors not captured in 2D assays. Soft 3D microenvironments also reprogram cancer cell metabolism toward tumor-repopulating states, including cytosolic phosphoenolpyruvate carboxykinase (PCK1) upregulation and mitochondrial PCK2 suppression to remodel gluconeogenic and TCA fluxes under compliant ECM conditions (Li et al, 2015; Luo et al, 2017). Together, these findings reveal metabolic consequences of dimensionality, where lower confinement in compliant 3D matrices reduces the energetic cost of migration and can enhance proliferative capacity, underscoring the need for physiologically relevant culture systems to accurately study mechanometabolic phenomena.

The altered mechanics of the TME also influence cancer-stroma interactions, further driving metabolic adaptations (Muir et al, 2018). Mechanical stress from matrix stiffening impairs perfusion, inducing hypoxia within tumors (Stylianopoulos et al, 2012), and amplifying the Warburg effect (Fu et al, 2023; Tian et al, 2020; Wise et al, 2011; Yoo et al, 2020; Zhang et al, 2024). Under hypoxia, HIF-1α downregulates mitochondrial phosphoenolpyruvate carboxykinase (PCK2), attenuating TCA cycle activity and causing fumarate accumulation and ROS production that promote tumor-repopulating cell growth (Tang et al, 2019). Hypoxic conditions driven by increased stiffness lead to HIF-1 stabilization (Cheu et al, 2023), which promotes metabolic rewiring and ECM remodeling by upregulating lysyl oxidase (LOX), a cross-linking enzyme that further stiffens the matrix (Schietke et al, 2010). LOX can also enhance HIF-1α protein synthesis by activating PI3K-Akt signaling (Pez et al, 2011), establishing a HIF-1/LOX feed-forward loop that reinforces TME stiffness and aggressiveness. Together, emerging evidence indicates that targeting mechanotransductive pathways can both curb therapeutic resistance and normalize the abnormal mechanics of the tumor microenvironment to aid in perfusion and drug response. Accordingly, treatment strategies should integrate mechanical interventions with conventional molecular therapies to more effectively limit tumor progression. Together, emerging evidence indicates that targeting mechanotransductive pathways can both curb therapeutic resistance and normalize the abnormal mechanics of the tumor microenvironment to aid in perfusion and drug response, highlighting the intimate relationship between mechanical cues and treatment efficacy. These same mechanical inputs modulate drug sensitivity by altering metabolic gene expression and nutrient signaling pathways, where compressive forces enhance PI3K activity and metabolic gene programs in breast and pancreatic cancer models (Di-Luoffo et al, 2025). In addition, marked differences in drug efficacy are observed between 2D and 3D cultures for agents such as doxorubicin and paclitaxel (Ikari et al, 2021). Accordingly, treatment strategies should integrate mechanical interventions with conventional molecular therapies to more effectively limit tumor progression. These observations suggest that metabolic inhibitors screened under static or 2D conditions may underestimate therapeutic vulnerabilities present in mechanically stressed microenvironment of tumors.

## Conclusion

Mechanical cues and cellular metabolism are fundamentally linked through multiple overlapping pathways, including cytoskeleton-mediated mechanotransductive responses. Mechanical cues from ECM stiffness, fluid shear stress, and cellular adhesions have been shown to influence key metabolic pathways, such as glycolysis and OXPHOS, primarily through alterations in the cytoskeleton. Cellular metabolism shapes various processes integral to mechanical behavior, such as cytoskeletal dynamics and force generation. This reciprocal interaction underlines the fundamental role of mechanometabolism in maintaining cellular homeostasis. Altered mechanical cues within the microenvironment profoundly affect cellular metabolism, providing new insights into the pathogenesis of diseases such as cancer, fibrosis, and cardiovascular disorders.

Despite notable progress in this field, gaps still exist in our understanding of the precise mechanisms by which mechanical signals are transduced into metabolic changes within cells. Further research is necessary to clarify the specific signaling pathways and molecular interactions that mediate the crosstalk between mechanical stimuli and cellular metabolic responses, as well as to explore the implications of these interactions in various cellular processes and diseases (Green et al, 2014; Ibar and Irvine, 2020). Mechanometabolism is embedded within developmental biology, regenerative medicine, cancer biology, and tissue engineering, and as such, research in this area could have a broad-reaching impact.

Stiffened ECMs, disrupted cell adhesion, and abnormal fluid dynamics contribute to metabolic reprogramming, driving disease progression and resistance to therapy. Mechanoregulation of cell metabolism enables cancer cells to adapt their metabolic pathways, allowing them to navigate the mechanical heterogeneity of the tumor microenvironment and ultimately escape the primary tumor to metastasize to other, mechanically distinct parts of the body. Similarly, ECM softening in fibrotic tissues shifts cells toward catabolic states, exacerbating tissue dysfunction. The dynamic interplay between ECM remodeling and cellular metabolic responses further highlights the intricate relationship between mechanical forces and metabolic pathways. ECM perturbation leads to metabolic changes that drive the cells towards a more malignant state. This understanding underscores the importance of targeting mechanotransduction pathways to restore metabolic equilibrium. Inhibiting YAP/TAZ, PI3K, or mTOR offers promising strategies for mitigating these metabolic vulnerabilities and enhancing therapeutic efficacy.

The dynamic interplay between mechanics and metabolism presents numerous open questions and exciting opportunities for future research. With the advent of new technologies that enable the study of metabolism, the emerging field of mechanometabolism is advancing rapidly. However, advancing our understanding will require tools capable of precisely manipulating mechanical forces while enabling metabolic analysis with single-cell resolution. Mechanometabolic responses are not uniform across a population. Distinct subpopulations can differ in cytoskeletal organization, force generation, and mitochondrial content, leading to divergent metabolic strategies even under identical mechanical cues. This mechanometabolic heterogeneity is magnified in steep mechanical gradients, such as the TME, where stiffer niches preferentially promote glycolytic metabolism. This resulting metabolic heterogeneity can be interrogated using single-cell metabolomics approaches such as matrix-assisted laser desorption/ionization mass spectrometry (MALDI-MS) and laser ablation electrospray ionization mass spectrometry (LAESI-MS) (Saunders et al, 2023). Complementary live-cell imaging innovations, including fluorescence-lifetime imaging microscopy (FLIM) and fluorescent reporter probes (Boone et al, 2026), enable dynamic, spatiotemporal resolution of metabolic states in intact cells. Complementary functional and tracer-based approaches, including single-cell energetic metabolism by profiling translation inhibition (SCENITH) (Argüello et al, 2020) and isotope-tracing microscopies, further resolve single-cell energetic dependencies and metabolic fluxes (Box 1). While much progress has been made in studying cell–ECM interactions, other mechanical stimuli remain comparatively underexplored. Furthermore, most studies have focused on isolated metabolic rewiring or regulatory mechanisms, with less emphasis on their broader physiological relevance. Recent innovations in tissue engineering technologies and 3D culture systems have begun to unveil novel metabolic behaviors in mechanically complex environments (Agarwal et al, 2017; Heaster et al, 2020; Janská et al, 2021; Kim et al, 2019b).

The dynamic interplay between mechanics and metabolism presents numerous open questions and exciting opportunities for future research. With the advent of new technologies that enable the study of metabolism, the emerging field of mechanometabolism is advancing rapidly.

Integrating mechanotransduction with metabolic pathways offers a promising avenue for therapeutic intervention, particularly in the context of targeted drug delivery and precision medicine. Combining 3D engineered ECMs with controlled mechanical stimulation enables a more physiologically relevant recapitulation of in vivo conditions. Coupling these systems with metabolic drug screening and single-cell metabolic readouts provides a powerful framework to predict context-dependent treatment responses and uncover mechanometabolic vulnerabilities that are obscured in conventional culture models. Looking forward, key challenges include defining how mechanical cues dynamically reprogram metabolic pathways across cell states and disease contexts and determining how these mechanometabolic phenotypes can be exploited to improve therapeutic stratification and efficacy (Box 1).

Box 1: In need of answers

- What is the cause of mechanometabolic heterogeneity at the single-cell level?
  - Cells exposed to identical mechanical cues can exhibit divergent metabolic states, likely arising from differences in cytoskeletal organization, mitochondrial content, and transcriptional regulation. Resolving how these heterogeneous states emerge and whether they are stably inherited will require integration of single-cell metabolomics, live-cell metabolic imaging, and lineage-resolved approaches.

- How do mechanical cues regulate metabolic plasticity across different microenvironmental contexts (2D vs 3D, physiological vs pathological)?
  - Mechanometabolic responses differ substantially between 2D and 3D systems, as well as between physiological and pathological environments. Addressing this will require engineered ECM systems and organotypic models that recapitulate in vivo mechanical complexity.

- Can mechanometabolic vulnerabilities be therapeutically targeted?
  - Mechanical cues shape metabolic vulnerabilities that are often obscured under static or reductionist culture conditions. Identifying and targeting these dependencies will require drug screening platforms that incorporate physiologically relevant mechanical inputs alongside metabolic readouts.

### Graphics

Figures 1 and  2 were created with a licensed version of BioRender.com.

## Supplementary information

Peer Review File
