# Supplementary material for: Cellular mechanometabolism: stimuli and implications
Source: EMBO Rep. 2026 May 9;27(11):2853–64. doi: 10.1038/s44319-026-00795-4 (PMC13260949; doi:10.1038/s44319-026-00795-4)
Supplement: Supplementary file 1 — Peer Review File [file 44319_2026_795_MOESM1_ESM.pdf]

EMBO reports

Cellular Mechanometabolism: Stimuli and Implications

Ismael Ortiz, Santiago Lopez, Raghu Kondapaneni, Jose Hernandez, Keefer Boone, and Cynthia Reinhart-King

Corresponding author(s): Cynthia Reinhart-King (cr73@rice.edu)

Review Timeline:

Submission Date:	15th Oct 25
Editorial Decision:	15th Dec 25
Revision Received:	15th Mar 26
Accepted:	14th Apr 26

Editor: Kurt Weir

Transaction Report:

(Note: With the exception of the correction of typographical or spelling errors that could be a source of ambiguity, letters and reports are not edited. Depending on transfer agreements, referee reports obtained elsewhere may or may not be included in this compilation. Referee reports are anonymous unless the Referee chooses to sign their reports.)

Dear Dr. Reinhart-King,

Thank you once more for the submission of your review to EMBO Reports. I have now received a full set of referee reports that are copied below. As you will see, the referees state that your manuscript is interesting and timely, but they have also several suggestions to improve it, which I kindly ask you to address. Of repeated note was the need for a greater discussion of the contradictory results seen for fiber alignment.

Please also address the following editorial points:

- Please add a 'Disclosure and competing interests statement'. For more information see https://link.springer.com/journal/44319/submission-guidelines#conflictsofinterest

- Please add up to five keywords to the title page.

- CRediT has replaced the traditional author contributions section because it offers a systematic machine readable author contributions format that allows for more effective research assessment. Please remove the Author Contributions section from the manuscript and use the free text boxes beneath each contributing author's name in our online systems to add specific details on the author's contribution. More information is available in our guide to authors.

- When returning a revised version, may I also kindly ask you to include a box called "In need of answers" that briefly outlines the major questions that are still open in the field in the form of a few bullet points. These questions can be accompanied by a brief explanation of what would be needed to address them and may prove helpful towards setting the stage for future experimentation in the field.

- When submitting your revised manuscript, we will require a Microsoft Word file (.docx) of the revised manuscript text including detailed figure legends (at the very end), but without the figures.

- Please provide the final figures as separate, high resolution files as .pdf, .eps, .tif, or .jpg (one file per figure). Please finalize the drafts provided and make sure they accurately illustrate the key scientific concepts that you wish to show.

Please also note the following general advice on review figures:

- If there are certain aspects of your figure draft that are based upon assumptions or where the scientific data remains ambiguous (for example, schematically depicting a presumed direct protein-protein interaction, protein shape or subcellular localizations etc.) please add a comment so that we can work with you on an accurate depiction. Please ensure the directionality and nature of interactions is presented accurately.

- If the figure or single panels of the figure have been adapted from a published figure, please add this information to the figure legend (e.g., 'Adapted from...' or 'Based on...'). The editor will discuss if a reference and permission will be necessary

- Please only re-use figures or parts of a figure if this is essential for understanding the concept communicated. Often a reference to a previous paper will suffice. If the figure contains re-used images or elements of images, including schematics, micrographs or photos, please make sure that you have the permission/license to publish it (this also applies to your own previous work, if the journal you published in retains copyright. Certain 'creative commons' open access licenses, such as CC-BY 4.0, allow re-use without additional formal permissions). All re-used material must be explicitly cited.

- If you use an image data base for scientific iconography (e.g., BioRender), please let us know if you have a license that allows for publication in an academic journal. Often authors use misleading iconography for expedience. Please ensure the information shown is scientifically accurate. If in doubt, please discuss with the editor.

Thank you again for writing this article for us. I look forward to seeing a revised version of your manuscript when it is ready. As for timing, would it be possible for you to submit the revised version by end of March? But let me know if you need more time.

Best regards,

Kurt Weir
Editor
EMBO Reports

Referee #1:

In this review, Ortiz et al summarized recent advances that reveal that mechanical cues in the microenvironment play a role in cellular metabolism. They discussed mechanical forces and cell-ECM interactions influences metabolic pathways and ATP production. While the topic is interesting, the manuscript in its present form suffers from insufficient description and analysis of the published findings, lack of discussion of potential mechanisms underlying the published results and missing some citations of literature.

Specific major comments:

1. The conventional view is that metabolism is dominated by biochemical processes and signaling pathways. However, over the last decades, increasing evidence suggest that mechanical signals also regulate cellular metabolic pathways. The authors in this manuscript highlighted some of the latest findings and understandings on this important function of the cells. However, the authors appear to focus on listing of individual articles without delving into some details of what the main findings mean. For example, in line 43, the authors stated that "Many of the classically identified mechanosensitive proteins are also metabolic effectors." without providing concrete evidence to support this claim.

2. Section II. ECM as a physical cue for metabolism regulation. The authors stated numerous times that "filamentous actin (F-actin), microtubules, and intermediate filaments (IFs), have also
been implicated in metabolic processes". This is a weak statement that uses the word "implicated". It is not clear if the authors would like to suggest that the cytoskeleton changes are correlated with metabolic processes or control or regulate metabolic processes?

3. A review in the same topic was published in 2021 at Nat Rev Mol Cell Biol (PMID: 33188273). The authors did not cite this review in this manuscript. The authors need to focus on the recent original publications since the 2021 review and provide analysis of these original articles and provide new insights on the topic of mechanical cues in the microenvironment regulating metabolism.

4. Line 67, "Given that the connection between the ECM and cells is mediated by the cytoskeleton". This is an imprecise statement, missing the adhesion molecules on the plasma membrane and the proteins that link the integrins with the cytoskeleton. This sentence needs to be revised.

5. Line 127-130, the authors list articles that appear to show opposite effects of fiber alignment. The authors suggested that this "differences in metabolic response may also be ECM132
type dependent", they did not discuss the possibility of plasma membrane alignment vs cytoskeletal alignment contributing to the opposite response from the same type of cells.

6. E. Mechanometabolism in the tumor microenvironment. In this section, the authors did not discuss the differences between the cells plated on 2D surfaces and the cells embedded within 3D matrices. The ECM stiffness would exert dramatically different effects on the cells and even opposite effects on cell invasion and proliferation. See published papers that soft 3D tumor stroma/matrix promotes cancer cell invasion and mitosis/proliferation and metastasis: PMID: 32905405; PMID:32749238; PMID:39975263; PMID:24981707. The authors need to clarify for the readers these differences of 2D vs 3D matrix and the existing explanations or models on the topic.

7. Fig. 1 is too general. Another figure with more specific pathways and analysis of the main findings of the latest discoveries since 2021 should be listed.

8. These missing references should be cited: 1) PCK1 upregulation in soft ECM in cancer stem cells. Cancer Res 201510.1158/0008-5472.CAN-14-2615
2) PCK2 downregulation in soft cancer stem cells in soft TEM. Oncogene 2017. DOI: 10.1038/onc.2016.520

3) hypoxia-induced attenuation of tricarboxylic acid (TCA) to promote growth of tumorigenic cells. Tang K et al Oncogene 2019. DOI: 10.1038/s41388-019-0932-1

4) Cadherin-AMPK on metabolism: NCB 2017. doi: 10.1038/ncb3537

5) a review paper; metabolism regulates stem-like phenotype, and epigenetic modifications of chromatin that drive neoplastic transformation. Saggese P et al Cancers 2020. DOI: 10.3390/cancers12123788

Minor point:

9. lines 31 and 35: Zhou et al, 2012; Zhao et al, 2024. These papers cannot be found in the references list.

Referee #2:

This MS provides a comprehensive and timely review of mechanometabolism, integrating recent findings on how mechanical cues shape cellular metabolic programs. The topic is important, well-aligned with current trends in mechanobiology, and the manuscript is rich in citations. The writing is generally clear, and the review perfectly bridges mechanobiology with emerging metabolic insights.
However, there are several areas where the manuscript would benefit from improved conceptual framing, some clarifications, and a more balanced interpretation of contradictory results, before considering publication.

Major Comments:

1) Although the manuscript covers many mechanometabolic mechanisms, the overarching conceptual framework is not fully articulated. For example, early in the "Introduction", it would be helpful to define what mechanometabolism is in concise terms.
2) Several sections describe associations between mechanical cues and metabolic outputs, but the causal pathways remain vague. For example, in Section II.A, it is described that changes in ECM architecture "reshape metabolism," but the molecular intermediates (e.g., integrin signaling, cytoskeletal restructuring) are not always explicitly connected.
In the shear stress section, it is unclear whether metabolic changes are primary responses to mechanosensing or secondary to altered signaling pathways such as eNOS/NO. Adding sentences explicitly distinguishing what is known to be causal vs. correlative will make the review more rigorous.
3) In the collagen alignment section, the manuscript mentions contradictory data between Zanotelli and Pickett, attributing differences to ECM type. This is a valuable point, but the discussion could be expanded. Developing this discussion would significantly strengthen this section.
4) The authors mention single-cell needs in the Conclusion, but never define: i)how mechanometabolism varies across cell subpopulations; ii)how spatially heterogeneous mechanical environments create metabolic gradients; iii)whether recent single-cell metabolomics or live-cell metabolic imaging tools (FLIM, SCENITH, isotope tracing microscopies) can probe mechanometabolic heterogeneity. This is an important and rapidly emerging area deserving more attention.
5) While the discussion on TME stiffness, YAP/TAZ, and HIF-1 is strong, it would benefit from including i) other stiffness-induced oncogenes in metabolic rewiring, including p53 mutations (PMID: 37880212; PMID: 29255172); ii) the role of interstitial flow, which is mentioned but not deeply discussed; iii) potential therapeutic failures when metabolic targeting ignores the mechanical context. Expanding these points would produce a more nuanced synthesis.
6) In the section "Mechanometabolism in the tumor microenvironment", the authors should also cite a recent review that includes these aspects (PMID: 37597464).

Minor Comments
1) Some terms are introduced without an initial definition (e.g., IFs, TME), although they are well-known. Consider defining upon first use.
2) A few sentences could be smoothed for grammar or clarity.
3) The figures should be referenced more explicitly within the text.
4) Be consistent with "OXPHOS," "oxidative phosphorylation," and similar terms.

Referee #3:

The authors provide a comprehensive and well-referenced review on mechanometabolics. While the breadth of coverage is commendable, the manuscript would benefit from a clearer and more focused central message. At present, the key takeaway of the review and its distinction from existing literature in the field are not fully apparent.
Although I appreciate the authors' intent to maintain a broad perspective, the inclusion of multiple cell types and discussion of both healthy and diseased states, sometimes within the same section, dilutes the overall focus. Narrowing the scope to a specific cell type or disease context would enhance clarity and thematic coherence.
In addition, while the review provides substantial discussion on YAP/TAZ signaling, it would benefit from the inclusion of Piezo1 and Piezo2, which are also central mechanosensors relevant to mechanometabolic regulation.
To further strengthen the conclusion section, the authors should incorporate a forward-looking perspective on the field, highlighting emerging questions and potential applications of mechanometabolic insights across disciplines.
Finally, the manuscript currently includes only one schematic. Adding additional figures to illustrate key concepts, along with tables summarizing major findings, would greatly enhance organization, clarity, and overall reader engagement.

In this review, Ortiz et al summarized recent advances that reveal that mechanical cues in the microenvironment play a role in cellular metabolism. They discussed mechanical forces and cell-ECM interactions influences metabolic pathways and ATP production. While the topic is interesting, the manuscript in its present form suffers from insufficient description and analysis of the published findings, lack of discussion of potential mechanisms underlying the published results and missing some citations of literature.

Specific major comments:

1. The conventional view is that metabolism is dominated by biochemical processes and signaling pathways. However, over the last decades, increasing evidence suggest that mechanical signals also regulate cellular metabolic pathways. The authors in this manuscript highlighted some of the latest findings and understandings on this important function of the cells. However, the authors appear to focus on listing of individual articles without delving into some details of what the main findings mean. For example, in line 43, the authors stated that "Many of the classically identified mechanosensitive proteins are also metabolic effectors." without providing concrete evidence to support this claim.

We agree there is a need for concrete evidence in introductory claims of the manuscript and the need to better integrate the findings across different studies. In what was previously line 43, we provided evidence and deeper detail into how canonical mechanosensitive proteins are active metabolic effectors (i.e. f-actin, YAP/TAZ) to elucidate the bidirectional crosstalk between mechanotransduction and cellular metabolism. Furthermore, we expanded on details from previous studies in the following paragraphs and connected the findings to better support our conclusions, rather than simply listing out prior work in isolation from one another.

2. Section II. ECM as a physical cue for metabolism regulation. The authors stated numerous times that "filamentous actin (F-actin), microtubules, and intermediate filaments (IFs), have also
been implicated in metabolic processes". This is a weak statement that uses the word "implicated". It is not clear if the authors would like to suggest that the cytoskeleton changes are correlated with metabolic processes or control or regulate metabolic processes?

We thank the reviewer for highlighting the ambiguity in our original wording regarding the role of the cytoskeleton in metabolic processes. We agree that the term “implicated” did not clearly convey whether cytoskeletal dynamics are merely correlated with or actively regulate cellular metabolism. To address this, we have revised the paragraph to explicitly reflect the regulatory and mechanistic role of cytoskeletal networks in metabolic control. The updated text now emphasizes that F-actin, microtubules, and intermediate filaments not only respond to cellular energy status but also actively influence metabolic processes through spatial organization of glycolytic enzymes and modulation of mitochondrial function. This

revision clarifies that cytoskeletal remodeling serves as a functional mediator linking ECM-derived mechanical cues to metabolic regulation, rather than a passive association.

3. A review in the same topic was published in 2021 at Nat Rev Mol Cell Biol (PMID: 33188273). The authors did not cite this review in this manuscript. The authors need to focus on the recent original publications since the 2021 review and provide analysis of these original articles and provide new insights on the topic of mechanical cues in the microenvironment regulating metabolism.

We have now cited the recommended Nature Reviews Molecular Cell Biology article in the Introduction and clarified how our review complements this prior work. Specifically, we emphasize how discrete microenvironmental forces (e.g., matrix stiffening, confinement, fluid shear, compression) influence mechanometabolic regulation and pathophysiological progression, extending the thematic scope of the earlier review. We also highlight original research published in recent years to provide an updated synthesis aligned with the reviewer's recommendation.

4. Line 67, "Given that the connection between the ECM and cells is mediated by the cytoskeleton". This is an imprecise statement, missing the adhesion molecules on the plasma membrane and the proteins that link the integrins with the cytoskeleton. This sentence needs to be revised.

We agree that ECM signaling is not mediated solely by the cytoskeleton but rather involves integrins and associated adhesion proteins that physically link the extracellular matrix to intracellular cytoskeletal networks. Accordingly, we have revised the sentence to explicitly include these adhesion molecules and their role in mechanochemical signal transmission. The updated text now reads: "Given that ECM-derived mechanical and biochemical cues are transmitted into the cell via integrins and associated adhesion proteins that link the ECM to the cytoskeleton." This revision improves mechanistic accuracy and clarity.

5. Line 127-130, the authors list articles that appear to show opposite effects of fiber alignment. The authors suggested that this "differences in metabolic response may also be ECM132 type dependent", they did not discuss the possibility of plasma membrane alignment vs cytoskeletal alignment contributing to the opposite response from the same type of cells.

After closely looking at the Pickett paper, we decided to remove it from our review. The reviewer is correct that cytoskeletal alignment likely plays a role. In the Pickett paper, they do not image the cells and fibers simultaneously, so it is not possible to say whether the alignment is sufficient to produce a cell response. There are no images of the cells in alignment and some of the images suggest that the cells are not responding to the alignment, an interesting finding but not one that the authors

explore. There are too many open questions and missing information to be able to draw strong conclusions here, and as such we decided not to comment on it.

6. E. Mechanometabolism in the tumor microenvironment. In this section, the authors did not discuss the differences between the cells plated on 2D surfaces and the cells embedded within 3D matrices. The ECM stiffness would exert dramatically different effects on the cells and even opposite effects on cell invasion and proliferation. See published papers that soft 3D tumor stroma/matrix promotes cancer cell invasion and mitosis/proliferation and metastasis: PMID: 32905405; PMID:32749238; PMID:39975263; PMID:24981707. The authors need to clarify for the readers these differences of 2D vs 3D matrix and the existing explanations or models on the topic.

We have now expanded the section on mechanometabolism in the tumor microenvironment to explicitly address the metabolic and behavioral differences observed in 2D versus 3D matrices. We incorporated discussion of how compliant 3D stromal environments reduce confinement and energetic demands to promote invasion and proliferation, while stiff 2D substrates elevate cytoskeletal tension and glycolytic flux. We also integrated the relevant literature cited by the reviewer (PMID: 32905405, 32749238, 39975263, 24981707) to clarify the mechanistic explanations and models underlying these contrasting responses.

Revision:

Added to Section E (“Mechanometabolism in the tumor microenvironment”), paragraph 4

7. Fig. 1 is too general. Another figure with more specific pathways and analysis of the main findings of the latest discoveries since 2021 should be listed.

We have now added another figure showing the specific pathways activated by fluid shear stress and cyclic stretch. Additionally, we have also strengthened the previous figure by including more pathways activated by ECM.

8. These missing references should be cited: 1) PCK1 upregulation in soft ECM in cancer stem cells. Cancer Res 201510.1158/0008-5472.CAN-14-2615
2) PCK2 downregulation in soft cancer stem cells in soft TEM. Oncogene 2017. DOI: 10.1038/onc.2016.520

We have now incorporated both references into the section discussing soft 3D tumor microenvironments and metabolic plasticity. Specifically, we added a sentence highlighting how compliant matrices induce PCK1 upregulation and PCK2

suppression to reprogram gluconeogenic and TCA metabolism in tumor-repopulating cells. This addition strengthens our discussion of how soft ECM niches support stem-like tumor growth through metabolic rewiring.

Revision:

Added to Section E ("Mechanometabolism in the tumor microenvironment"), paragraph 4, prior to the concluding synthesis sentence.

3) hypoxia-induced attenuation of tricarboxylic acid (TCA) to promote growth of tumorigenic cells. Tang K et al Oncogene 2019. DOI: 10.1038/s41388-019-0932-1

We thank the reviewer for pointing us to this important study. We have now cited Tang et al. (2019) and added text describing how hypoxia-driven downregulation of PCK2 attenuates TCA cycle activity, leading to fumarate accumulation and ROS production that support tumor-repopulating cell growth. This strengthens our discussion of hypoxia-induced metabolic reprogramming within the tumor microenvironment.

Revision:

Added to Section E ("Mechanometabolism in the tumor microenvironment"), paragraph on hypoxia-driven metabolic adaptation.

4) Cadherin-AMPK on metabolism: NCB 2017. doi: 10.1038/ncb3537

We agree that this is an excellent paper, but we struggled to find a section within our review where it fits well. Since we largely focus on exogenous forces, we felt it did not naturally fit within the scope of our review.

5) a review paper; metabolism regulates stem-like phenotype, and epigenetic modifications of chromatin that drive neoplastic transformation. Saggese P et al Cancers 2020. DOI: 10.3390/cancers12123788

We thank the reviewer for highlighting this relevant review. We have now cited Saggese et al. (2020) and added text describing how metabolic rewiring regulates epigenetic modifiers and stem-like cancer phenotypes that contribute to neoplastic progression. This addition contextualizes metabolic plasticity within cancer identity and complements our discussion of mechanometabolic adaptations.

Revision:

Added to Section E (“Mechanometabolism in the tumor microenvironment”), immediately after the sentence discussing metabolic plasticity and before the introduction of the Warburg effect.

Minor point:

9. lines 31 and 35: Zhou et al, 2012; Zhao et al, 2024. These papers cannot be found in the references list.

Thank you for catching this error. The citations have been added to the reference list and appear in the antepenultimate and ultimate positions.

Referee #2:

This MS provides a comprehensive and timely review of mechanometabolism, integrating recent findings on how mechanical cues shape cellular metabolic programs. The topic is important, well-aligned with current trends in mechanobiology, and the manuscript is rich in citations. The writing is generally clear, and the review perfectly bridges mechanobiology with emerging metabolic insights.
However, there are several areas where the manuscript would benefit from improved conceptual framing, some clarifications, and a more balanced interpretation of contradictory results, before considering publication.

Major Comments:

1) Although the manuscript covers many mechanometabolic mechanisms, the overarching conceptual framework is not fully articulated. For example, early in the "Introduction", it would be helpful to define what mechanometabolism is in concise terms.

To improve clarity and guide the reader, we have added a concise definition in the Introduction that explicitly describes the integration of mechanotransduction and metabolic regulation. The revised text now includes a definition of mechanometabolism as referring to the integration of mechanotransduction and metabolic regulation, in which physical cues from the extracellular microenvironment are translated into intracellular signaling programs that reshape cellular metabolism. This addition helps establish a clear conceptual foundation for the mechanisms discussed throughout the manuscript.

2) Several sections describe associations between mechanical cues and metabolic outputs, but the causal pathways remain vague. For example, in Section II.A, it is described that changes in ECM architecture "reshape metabolism," but the molecular intermediates (e.g., integrin signaling, cytoskeletal restructuring) are not always explicitly connected.

In the shear stress section, it is unclear whether metabolic changes are primary responses to mechanosensing or secondary to altered signaling pathways such as eNOS/NO. Adding sentences explicitly distinguishing what is known to be causal vs. correlative will make the review more rigorous

We appreciate the reviewer's insightful comment regarding the causal relationship between shear stress and metabolic changes. To clarify this distinction, we revised the section to explicitly note that while shear stress influences metabolism, certain metabolic responses appear to be secondary to upstream signaling. Specifically, we now reference studies showing that silencing of metabolic regulators such as HIF-1α and AMPK can override the metabolic effects of oscillatory shear stress, reducing glycolysis and enhancing oxidative phosphorylation, thereby suggesting that mechanosensing alone is not sufficient to maintain a glycolytic phenotype. This addition helps distinguish direct mechanical effects from downstream signaling-mediated metabolic regulation. We also clarified some of the vague language mentioned in some sections.

3) In the collagen alignment section, the manuscript mentions contradictory data between Zanotelli and Pickett, attributing differences to ECM type. This is a valuable point, but the discussion could be expanded. Developing this discussion would significantly strengthen this section.

We thank the reviewer for this constructive suggestion. In response, we have expanded the collagen alignment section to more explicitly address how differences in ECM composition may underlie the apparently contradictory metabolic outcomes reported by Zanotelli et al. and Pickett et al. Specifically, we now emphasize that collagen is not a passive scaffold but a biologically active, viscoelastic, and degradable matrix that presents native integrin-binding ligands, whereas aligned synthetic fibers often differ substantially in stiffness, ligand presentation, and remodeling capacity.

4) The authors mention single-cell needs in the Conclusion, but never define:

i)how mechanometabolism varies across cell subpopulations;
ii)how spatially heterogeneous mechanical environments create metabolic gradients;-
iii)whether recent single-cell metabolomics or live-cell metabolic imaging tools (FLIM, SCENITH, isotope tracing microscopies) can probe mechanometabolic heterogeneity. This is an important and rapidly emerging area deserving more attention.

We have expanded the Conclusion to explicitly address mechanometabolic heterogeneity at the single-cell level. Specifically, we now (i) describe how mechanometabolic responses vary across cell subpopulations due to differences in cytoskeletal organization, force generation, and mitochondrial content; (ii) discuss how spatially heterogeneous mechanical environments, such as stiffness gradients

in the tumor microenvironment, amplify metabolic heterogeneity and bias metabolic pathway usage; and (iii) highlight recent and emerging single-cell approaches capable of probing this heterogeneity including FLIM, SCENITH, isotope tracing microscopies, and fluorescent probes. Together, these additions provide a clearer forward-looking perspective on how mechanometabolic heterogeneity can be experimentally resolved and leveraged in future studies.

5) While the discussion on TME stiffness, YAP/TAZ, and HIF-1 is strong, it would benefit from including i) other stiffness-induced oncogenes in metabolic rewiring, including p53 mutations (PMID: 37880212; PMID: 29255172); ii) the role of interstitial flow, which is mentioned but not deeply discussed; iii) potential therapeutic failures when metabolic targeting ignores the mechanical context. Expanding these points would produce a more nuanced synthesis.

We thank the reviewer for these insightful suggestions. We have addressed each point in the revised manuscript. First, we now discuss stiffness-dependent control of mutant p53, including its mechanical stabilization through a mevalonate–RhoA axis and its role in sustaining amino acid metabolism through serine–glycine synthesis and LAT1 upregulation. Second, we expanded our discussion of interstitial flow as a contributor to mechano-metabolic coupling within solid tumors. Third, we added closing text noting that mechanical context influences metabolic drug responses, and that therapeutic efficacy may be underestimated when screening metabolic inhibitors under static or 2D conditions. Together, these additions provide a more nuanced synthesis of mechanometabolic regulation within the tumor microenvironment and strengthen the translational implications of our review.

Revision Locations:

Section E: Mechanometabolism in the Tumor Microenvironment, after the stiffness→YAP/TAZ→mTOR discussion (mutant p53 addition)

Section E, immediately following the sentence describing mechanical cues in the TME (interstitial flow addition)

Section E, closing two sentences of the section (therapeutic/translation addition)

6) In the section "Mechanometabolism in the tumor microenvironment", the authors should also cite a recent review that includes these aspects (PMID: 37597464).

We have now cited the recommended review (PMID: 37597464) in Section E (“Mechanometabolism in the tumor microenvironment”) immediately after the line

stating that metabolic plasticity is actively directed by the TME via progressive matrix stiffening.

Minor Comments

1) Some terms are introduced without an initial definition (e.g., IFs, TME), although they are well-known. Consider defining upon first use.

We have defined IF and TME to clarify terminology, thank you for the guidance.

2) A few sentences could be smoothed for grammar or clarity.

We have corrected grammar mistakes and smoothed awkward phrasing.

3) The figures should be referenced more explicitly within the text.

Thank you for this suggestion, we have now referenced the figures within the text.

4) Be consistent with "OXPHOS," "oxidative phosphorylation," and similar terms.

Thank you for noticing this discrepancy, we now consistently use OXPHOS once it is defined.

Referee #3:

The authors provide a comprehensive and well-referenced review on mechanometabolics. While the breadth of coverage is commendable, the manuscript would benefit from a clearer and more focused central message. At present, the key takeaway of the review and its distinction from existing literature in the field are not fully apparent.

Although I appreciate the authors' intent to maintain a broad perspective, the inclusion of multiple cell types and discussion of both healthy and diseased states, sometimes within the same section, dilutes the overall focus. Narrowing the scope to a specific cell type or disease context would enhance clarity and thematic coherence.

We thank the reviewer for this thoughtful perspective. The inclusion of multiple cell types in both physiological and pathophysiological states was a deliberate choice intended to highlight shared and differing mechanistic principles across various systems rather than exhaustively focus on a single model. We believe this comparative framework strengthens the manuscript’s central theme and broadens its relevance. Reviewer 2 suggested expansion of discussion on various cell types which we have incorporated in the revision.

In addition, while the review provides substantial discussion on YAP/TAZ signaling, it would benefit from the inclusion of Piezo1 and Piezo2, which are also central mechanosensors relevant to mechanometabolic regulation.

We have now incorporated discussion of Piezo1 and Piezo2 as mechanosensitive ion channels relevant to mechanometabolic regulation. We added (i) contextual background on Piezo-mediated calcium influx and mechanotransduction, (ii) a brief section on Piezo1-dependent metabolic and immune signaling under fluid shear stress, and (iii) mechanistic and therapeutic implications of Piezo activity within the Fluid Shear Stress section. These additions expand the mechanosensor framework and address the reviewer's recommendation.

To further strengthen the conclusion section, the authors should incorporate a forward-looking perspective on the field, highlighting emerging questions and potential applications of mechanometabolic insights across disciplines.

To strengthen the forward-looking perspective of the Conclusion, we have expanded this section to highlight emerging experimental directions, open questions, and translational applications of mechanometabolic research. Specifically, we now emphasize how advances in 3D engineered extracellular matrix (ECM) platforms with controlled mechanical stimulation enable more physiologically relevant modeling of in vivo microenvironments, and how integration with metabolic drug screening and single-cell metabolic readouts provides a framework to uncover context-dependent mechanometabolic vulnerabilities. We further outline key open questions regarding how mechanical cues dynamically reprogram metabolic pathway usage across cell states and disease contexts and discuss how these insights may inform therapeutic stratification and precision medicine efforts across oncology and other mechanically regulated systems.

Finally, the manuscript currently includes only one schematic. Adding additional figures to illustrate key concepts, along with tables summarizing major findings, would greatly enhance organization, clarity, and overall reader engagement.

We thank the reviewer for this helpful suggestion. To improve reader engagement and illustrate key pathways, we have added another figure showing the specific pathways activated by fluid shear stress and cyclic stretch. Additionally, we have also strengthened the previous figure by including more pathways activated by ECM.

1st Revision - Editorial Decision **14th Apr 2026**

Cynthia Reinhart-King
Rice University
Bioengineering
United States

Dear Dr. Reinhart-King,

I am pleased to inform you that your manuscript has been accepted for publication in EMBO reports. Your manuscript will be processed for publication by EMBO Press. It will be copy edited and you will receive page proofs prior to publication. Please note that you will be contacted by Springer Nature Author Services to complete licensing information.

Should you be planning a Press Release on your article, please get in contact with embo_production@springernature.com as early as possible in order to coordinate publication and release dates.

If you have any questions, please do not hesitate to contact the Editorial Office. Thank you for your contribution to EMBO Reports.

Yours sincerely,

Kurt Weir
Editor
EMBO Reports

>>> Please note that it is EMBO Reports policy for the transcript of the editorial process (containing referee reports and your response letter) to be published as an online supplement to each paper. If you do NOT want this, you will need to inform the Editorial Office via email immediately. More information is available here: https://link.springer.com/partners/embo-press/editorial-policies#Peer%20review
